# Defining the human lung pathodegradome of the V8 protease from *Staphylococcus aureus*

**DOI:** 10.1128/iai.00725-25

**Published:** 2026-03-05

**Authors:** Emilee M. Mustor, Dale Chaput, Sophie Sipprell, Lindsey N. Shaw

**Affiliations:** 1 Department of Molecular Biosciences, University of South Florida 7831, Tampa, Florida, USA; 2 Center for Antimicrobial Resistance, University of South Florida 7831, Tampa, Florida, USA; University of California Davis, Davis, California, USA

**Keywords:** *Staphylococcus aureus*, TAGS-CR, V8 protease, human lungs, pathodegradome

## Abstract

*Staphylococcus aureus* is a globally relevant human pathogen and key driver of human morbidity and mortality. During infection, it employs a suite of extracellular proteases to subvert host defenses and promote disease progression. Key among these, the V8 serine protease (SspA) stands out as a master manipulator of the immune response; however, host targets for this enzyme remain incompletely defined. Herein, we use a high-efficiency N-Terminomic approach developed by our group (TAGS-CR) to capture ~320 biologically relevant V8 targets in the human lung proteome. These substrates present a role for V8 in modulating the complement system, with specific targets like Complement C3 carrying significant importance in this niche. In addition, neutrophil effector functions such as ROS production, migration, phagocytosis, and degranulation were disrupted by the action of V8. Similarly, epithelial barrier integrity may suffer as a consequence of V8 targeting foundational proteins, such as zona occludens and alpha actinin proteins, coupled with supporting actin molecules. Notably, our data provide new insights into V8 as a key player exploiting nutritional immunity by disrupting iron-complexed proteins such as transferrins, hemopexin, and hemoglobin. In addition to its capacity to intensify inflammation, V8 also undermines the function of key immune regulators, including pro-inflammatory sink molecules like gelsolin. Collectively, this comprehensive proteolytic mapping expands our understanding of how *S. aureus* can manipulate human biology across infection-relevant niches and offers valuable insight into conserved molecular mechanisms that underlie bacterial virulence.

## INTRODUCTION

The extracellular proteases of *Staphylococcus aureus* are key factors driving the pathogenesis of this dangerous human microbe (1, 2). Included in its arsenal of secreted virulence determinants, *S. aureus* possesses myriad such enzymes, including a metalloproteinase (Aureolysin, *aur*), two cysteine proteases (Staphopain A [*scpA*] and Staphopain B [*sspB*]), a glutamyl endopeptidase (*sspA* or the V8 serine protease), and depending on the sequence type, up to nine serine-protease-like enzymes (3, 4). Over time, their roles during infection have progressively become more clear: On the one hand, they control the stability of self-derived virulence factors, while, on the other, degrading host proteins and dysregulating the immune response (1, 2). Toward this latter role, it has become increasingly important to identify protease substrates so as to understand how these enzymes promote bacterial dissemination, immune evasion, and tissue damage, particularly in the context of differing infection types.

Manifestations of *S. aureus* disease range from skin and soft tissue infections to more severe invasive conditions, such as bacteremia, endocarditis, osteomyelitis, and pneumonia (5, 6). Given that each infectious niche requires different bacterial adaptations, capturing protease substrates within these settings could reveal unique targets for the development of new antimicrobial therapies. With that said, it is true that many protease substrates are applicable to all infection types, for example, cleavage of neutrophil proteins, such as CXCR2, CD31, galectin-3, and antimicrobial peptide LL-37 (7–10). Additionally, targeting of protease inhibitors, such as alpha-1-protease inhibitor and alpha-1 anti-chymotrypsin, immunoglobulins, and modulation of the clotting cascade and complement system, may result in general protease-induced dysregulation of the host immune response during disseminated infection (7–11). However, it is also true that individual proteases have been implicated in specific disease types. For example, tissue injury due to collagen cleavage by ScpA and SspB, in combination with elastin degradation by ScpA, has been suggested to influence the progression of infective endocarditis (12, 13). Additionally, the cysteine protease ScpA has been shown to induce vascular leakage (VL) via the release of bradykinin from kininogen, a phenomenon enhanced by the activity of SspB (14). Moreover, when functioning in concert, they release an additional novel kinin shown to have similar effects (14). While VL in this context was found primarily as a consequence of cysteine protease activity, notably the release of bradykinin has also been observed for the V8 protease (15). V8 has a well-recognized role in atopic dermatitis and epithelial barrier disruption (16, 17). While this protease has been shown to alter important barrier proteins like ZO-1, which it is hypothesized to redistribute or degrade, only claudin-1 and PAR1 have been confirmed as additional V8 substrates in this context (18, 19). Regarding *S. aureus* pneumonia, only SplA and ScpA have recognized roles, inducing mucin-16 and surfactant protein A degradation in the lungs, respectively (20). Proteases like the SplD, among other Spls, have been shown to contribute to airway disease, yet no substrates have yet been elucidated (21).

This limited library of protease substrates severely underrepresents the potential influence these virulence factors can have on infection dynamics and disease outcome. Thus, new approaches are necessary to achieve global substrate characterization. Our group was the first to present such a study for a bacterial protease, evaluating the V8 pathodegradome within human serum (22). This study revealed ~90 substrates and established that this protease can modulate the host clotting cascade, cleave a wealth of complement factors, and inactivate host protease inhibitors, among other host proteins relevant to pathogenesis (23). We recently extended these findings by developing a new method of N-Terminomics that has enhanced target identification: Terminal Amine Guanidination of Substrates-Charge Reversal (TAGS-CR) (23). TAGS-CR was applied to study the human neutrophil degradome of the V8 protease, revealing ~350 substrates that highlighted the influence of this enzyme on neutrophil migration, intracellular defense, phagocytosis, and apoptosis (23). Thus, global substrate identification can and has revealed the broader scope of protease influence during bacterial infection. Herein, we extend our existing work by mapping the human lung pathodegradome of V8. Through substrate profiling, we uncovered ~320 targets, suggesting a role for this enzyme in promoting inflammation, eliciting tight junction and epithelial barrier damage, facilitating iron scavenging, and perpetuating immune evasion. Our study not only continues the characterization of V8 targets in infection-relevant locales but also provides unparalleled insight into niche-specific protease substrates, potentially revealing key mechanisms for adaptive virulence, immune evasion, and unveiling novel therapeutic pathways.

## RESULTS AND DISCUSSION

### Profiling of V8 substrates within the human lung

**Fig 1:**
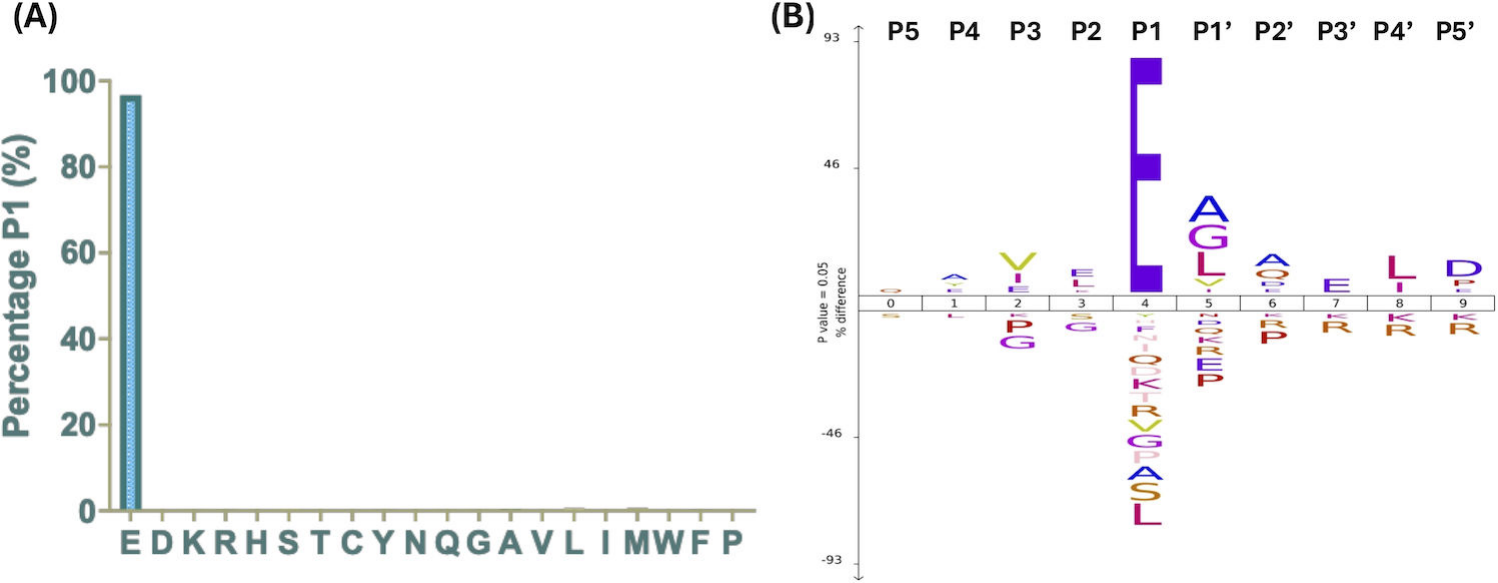
Sequence analysis validates V8 substrate specificity and cleavage events within the human lung proteome. Analysis of the amino acid identities surrounding cleavage sites of the 325 V8 human lung substrates. (**A**) Glutamic acid (E) constituted 99% of the amino acids at P1, directly in line with V8’s known substrate specificity. (**B**) ICELOGO analysis demonstrates the relative frequency of amino acids on either side of the site of proteolysis, denoted as P5-P5′.

We recently developed a new N-terminomic methodology for enhanced and efficient protease target identification: TAGS-CR (23). Herein, we leverage this method to elucidate the human lung substrate repertoire of the *S. aureus* V8 protease to provide further insight into its role in pathogenesis. As such, human lung tissue was homogenized and exposed to purified V8 protease. Subsequent mass-spectrometric and bioinformatic analysis comparing the V8 treated condition to untreated controls revealed 483 unique cleavage events in 325 human lung proteins (Tables S1 and S2). Importantly, the V8 protease has a unique substrate specificity, demonstrated in numerous studies by ourselves and others, cleaving on the carboxyl side of glutamic acid residues (E), although cleavage at aspartic acid residues (D) is also possible, but far less common (22, 24). Accordingly, initial validation of these 325 targets was achieved via sequence analysis of the amino acids surrounding the captured cleavage sites (Fig. 1A and B). We analyzed the percentage identity of amino acids at position P1, the carboxyl side of proteolysis, and found glutamic acid residues to account for the majority at ~99% (Fig. 1A). As an additional dimension of target validation, ICELOGO plots (25) were generated for the percentage difference of amino acids, relative to the human proteome, at the five positions flanking either side of the cleavage site (Fig. 1B). Unsurprisingly, only glutamic acid was found to be favored at P1, consistent with the substrate preference of V8 (Fig. 1B). Interestingly, cross-examination of this ICELOGO plot to that of our previous study investigating the V8 pathodegradome of human neutrophils (23) revealed striking similarities in the relative frequencies of amino acids at multiple positions (Fig. S1). On the C-terminal side of the cleavage site, favored amino acids included: alanine (A), glycine (G), leucine (L), valine (V), and isoleucine (I) at P1′; alanine (A) at P2′; leucine (L) and isoleucine (I) at P4′; and aspartate (D) and proline (P) at P5′(Fig. S1A and B). Furthermore, both studies demonstrated that proline (P) is not well tolerated at P1′-P2′, and that arginine (R) residues are similarly unfavored at P3′-P5′ (Fig. S1A and B). In keeping with this, amino acids found toward the N-terminus also revealed overlapping trends in both increased and decreased relative frequencies. ICELOGO plots showed a shared increase in the presence of leucine (L) and glutamic acid (E) at P2; valine (V) and isoleucine (I) at P3; and alanine (A) at P4, while glycine (G) and serine (S) are not well tolerated at P2; glycine (G) and proline (P) are similarly not favored at P3 (Fig. S1A and B). These findings strongly indicate a consensus sequence for the V8 protease beyond just that of glutamic acid (E) at the carboxyl side. To further bolster this idea, we compared both our human neutrophil pathodegradome (23) and the present human lung cleavage data to that of our previous study investigating human serum V8 substrates using the TAILS method of N-Terminomics (22) (Fig. S1A through C). Across all three studies, proline (P) is not tolerated at P1′-P2′, as is arginine (R) at P4′-P5′; while alanine (A), glycine (G), and valine (V) were all similarly increased in relative frequency at P1’ (Fig. S1A through C). Furthermore, all three studies observed glycine (G) and proline (V) to have a decreased frequency at P2 and P3, respectively, while an increased presence in valine at P3 was also maintained (Fig. S1A through C). This significant overlap in sequence specificity across multiple groups of infection-relevant substrates (human neutrophils, human lung tissue, and human serum) highlights a conserved targeting strategy of the V8 protease that appears to be stringently selective.

### The V8 protease cleaves proteins mediating host immune signaling pathways within human lungs

**Fig 2:**
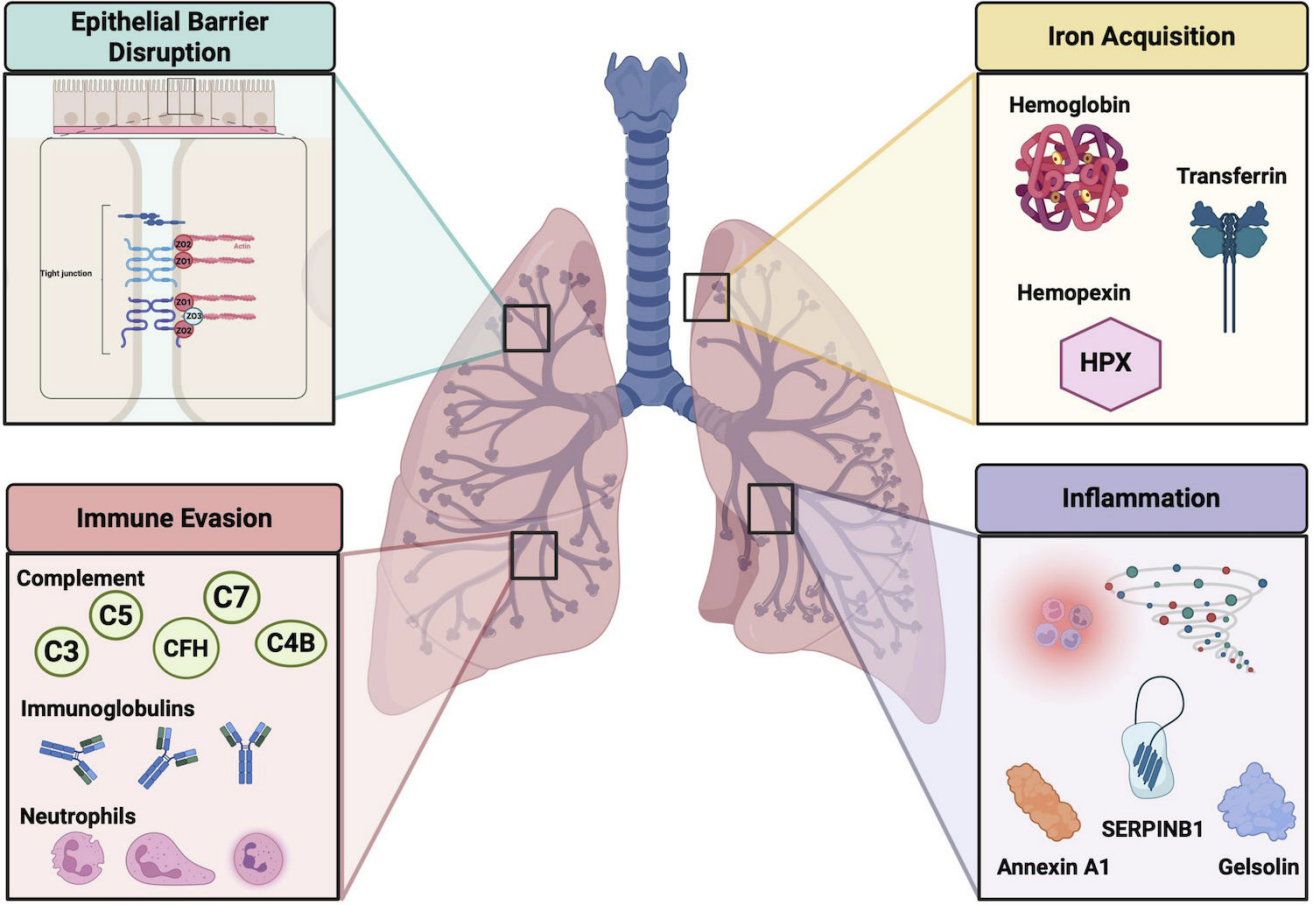
Infection-relevant host processes disrupted by the *S. aureus* V8 protease in human lung tissue. IPA of the 325 human lung V8 substrates revealed over 800 canonical signaling pathways can be modulated by this protease. We found V8 may contribute to immune evasion via cleavage of complement factors, immunoglobulins, and neutrophil proteins. Relevant to *S. aureus* pathogenesis, IPA also revealed V8 may play a role in epithelial barrier disruption, iron acquisition within the host, and the promotion of inflammation.

Enzymatic manipulation of host-signaling cascades is a foundational strategy for bacterial survival and persistence within the host (26). As such, target identification has enabled us to further describe host-pathogen interplay by uncovering key cellular processes being disrupted or exacerbated by protease activity. Ingenuity pathway analysis (IPA) of our human lung V8 substrates revealed over 800 signaling cascades that may experience proteolytic interference during infection, with the major themes summarized in Fig. 2. Unsurprisingly, given that neutrophils are recognized as first responders to infection and are key immune cells defending against *S. aureus* (27), the neutrophil degranulation pathway was found to possess the highest number of protein targets, 48, cleaved by V8 (Table S3). Beyond degranulation, a multitude of other neutrophil pathways, such as leukocyte extravasation, integrin signaling, neutrophil extracellular trap, and fMLP signaling in neutrophils, were also revealed by IPA to be modulated by V8 (Table S3). The ability of V8 to target neutrophil functionality on many different levels is documented in our previous study, which also captured these pathways (23). These overlapping results bolster the validity of our studies and underscore the conserved ability of V8 to exploit important host defense tactics like immune cell function. In keeping with this, IPA analysis also detected V8 targets belonging to a variety of pathways involved in immune activation, including pattern recognition receptor signaling, MyD88 signaling, ILK signaling, RAF/MAP kinase cascade, PI3K/AKT signaling, and Protein Kinase A signaling. While tight control of inflammation is necessary to balance pathogen clearance in the host while minimizing tissue damage, infectious microorganisms like *S. aureus* can manipulate this fragile process for self-gain. Our data suggest this may be an additional mechanism of action for V8 when contributing to disease causation, but more so, may be applicable when addressing the dynamics of pulmonary infections. Furthermore, V8’s known ability to cause epithelial barrier disruption was also represented herein, with cleavage detected for pathways such as remodeling of epithelial adherens junctions, tight junction and gap junction signaling, and cell junction organization. This supports previous studies demonstrating impaired barrier integrity in airway epithelial cells (AECs) as a consequence of V8 action (16). Lastly, IPA analysis unveiled a variety of other canonical signaling pathways with direct relevance to *S. aureus* pathogenesis as being affected by this protease, including complement signaling, apoptosis, immunoglobulins, antigen presentation, phagocytosis and phagosome formation, coagulation cascades, and iron homeostasis.

### TAGS-CR reaffirms the role of V8 as a modulator of the complement system

The complement system is indispensable in its role in host defense, providing initial innate immune protection as well as humoral immunity. Thus, it is unsurprising there is long-standing knowledge of the complement system being adversely affected by the action of several *S. aureus* virulence factors, including the extracellular proteases (28–30). While complement factor substrates have previously been defined for Aureolysin and SplB, currently the most comprehensive list of targets belongs to that of V8, captured by our group using TAILS to study the human serum pathodegradome of V8 (9, 22, 31). In line with previous findings, herein we again captured cleavage of complement factors C3, C4-B, C5, CfH by V8 and identified C7 as a new target. Considering not only our use of varied N-terminomic strategies but also different infection-relevant sources, our data herein reinforce the notion that the complement system is markedly vulnerable to V8 activity.

**Fig 3:**
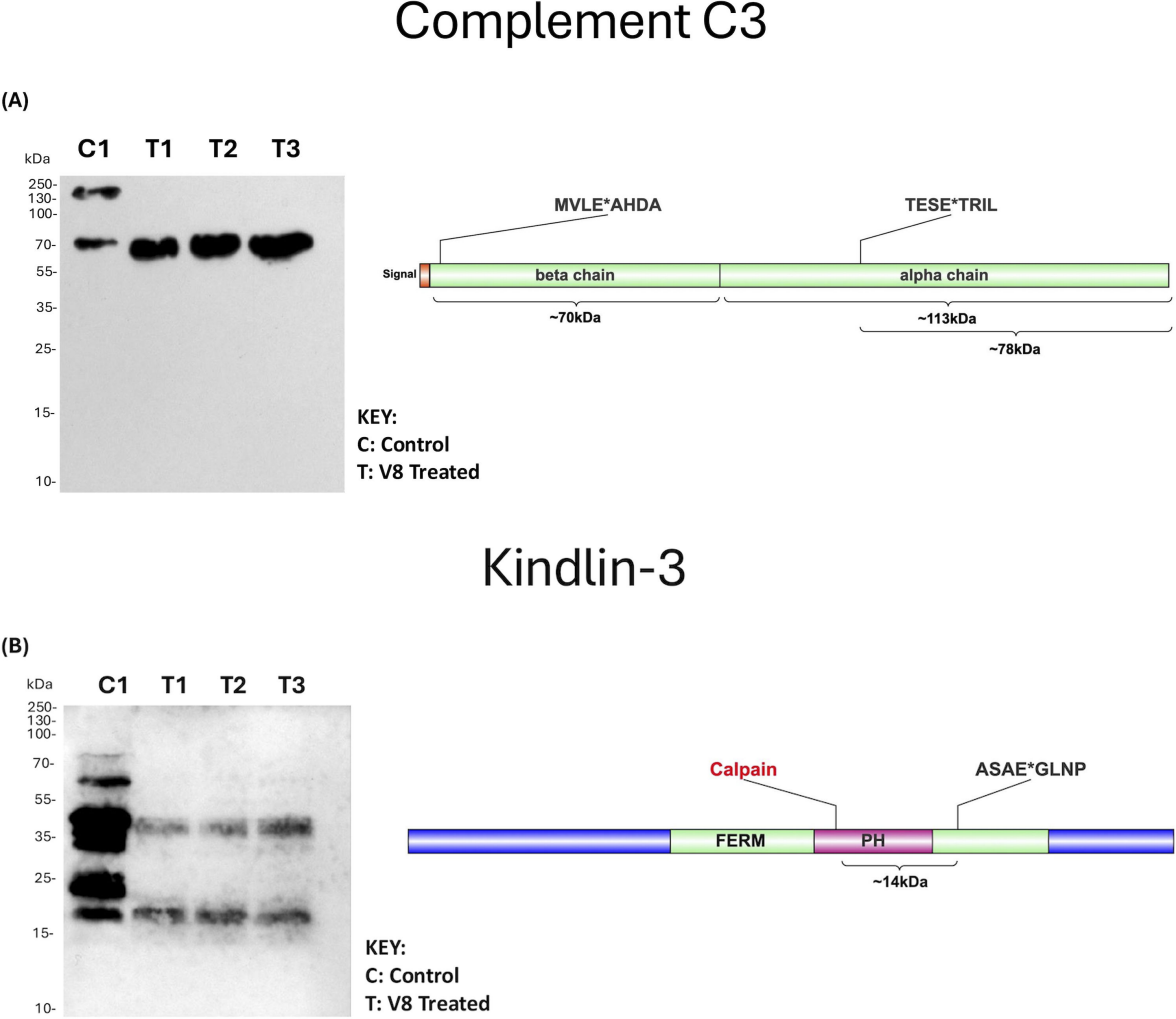
Western blot validation of human lung V8 targets. Homogenized human lung tissue was exposed to purified V8 protease from *S. aureus* at a concentration of 200 ng, while the control sample remained untreated. Proteomes were incubated overnight at 37°C. Degradation patterns corresponding to our N-terminomic data are shown, alongside amino acids surrounding the V8 cleavage site, denoted by *. (**A**) Complement C3; (**B**) Kindlin-3. FERM, Ferm domain. PH, PH domain. Calpain (red) indicates the predicted cleavage of Kindlin-3 by Calpain within neutrophil proteomes.

Importantly, cleavage of such factors by V8 is significant in the context of lung infections (32, 33) as, for example, C3 has been found to aid specifically in AEC protection in the presence of high stress conditions, such as oxidative stress, which commonly occurs during *S. aureus* infection (27, 32, 34). It has been shown that AECs can not only synthesize and secrete C3 but critically, they also possess C3 intracellular stores, hypothesized to provide rapid on-set protection during acute disease (34). It is noteworthy that V8 cleavage of C3 occurs at positions 46 and 978, both of which are located within the C3b molecule that promotes bacterial opsonization and clearance from the host (Table S2 and Fig. 3A) (35). Critically, V8 cleavage at position 978 occurs in the CUBg domain, which, in combination with the thioester-containing domain (TED) of C3b, has been found to maintain covalent interactions with pathogen cell surfaces (36). Interestingly, this cleavage event was also conserved in our V8 human serum study (22). Moreover, it is notable to mention that all complement V8 substrates identified herein, which include C3, C4b, C5, C6, C7, and CfH, were found in a previous study of *S. aureus* during the early stages of pneumonia infection (37)

An important aspect of any degradomic study is cleavage validation. Accordingly, we began this endeavor with complement C3 (Fig. 3A). This molecule possesses a beta chain (~70 kDa) as well as an alpha chain at (~113 kDa) (Fig. 3A). Physiological processing of this molecule is complex, whereby the C3a molecule from the alpha chain is first released, leaving behind C3b, which consists of fragments of both beta and alpha chains linked by disulfide bonds. C3b can be further cleaved *in vivo* to iC3b, followed by the release of C3f and then C3dg. In untreated human lung control samples, immunoblotting revealed intact complement C3 as well as a secondary fragment at ~70 kDa that either corresponds to the C3 beta chain or the alpha chain fragment of iC3b, both of which have similar molecular weights. In V8-treated samples, the full-length protein is clearly absent, and a single band is present just above 70 kDa that directly corresponds to the predicted fragment of the alpha chain following V8 cleavage (Fig. 3A).

### V8 targets neutrophil functionality within the lung environment

Further modulation of the innate immune system by V8 can be observed in our N-terminomic data via cleavage of critical neutrophil proteins. These immune cells remain steadfast as frontline defenders against *S. aureus* invasion, providing initial host protection via defensive strategies such as reactive oxygen species (ROS) production, phagocytosis, neutrophil extracellular trap (NET) formation, and degranulation (27). Unsurprisingly, IPA analysis identified a multitude of V8 substrates belonging to these pathways (Table S3) that are also captured in our V8-neutrophil study (23). Considering that defects in neutrophil functionality often lead to recurrent and fatal bacterial infections, such as chronic granulomatous disease (CGD), understanding how bacterial pathogens dysregulate their activity becomes critical in determining disease outcomes.

The most prominently affected signaling pathway was neutrophil degranulation, with a total of 48 proteins cleaved by V8, with several either directly or indirectly involved in the antimicrobial effector functions of these immune cells (Table S3). One example is lysosome-associated membrane protein-1, or LAMP-1, a host defense molecule part of neutrophil-specific or secondary granules (38). Indeed, LAMP1 is an essential protein for phagosome maturation, mediating their fusion with lysosomes necessary for pathogen clearance; and critically, phagosomes containing *S. aureus* have been shown to co-localize with LAMP1 (38, 39). V8 cleavage of this protein occurs at position 136 within the first luminal N-domain of LAMP1, found to be necessary for correct assembly of the functional multimeric complex (40). As such, LAMP1 deficiency due to V8 activity may assist in promoting intracellular survival as a consequence of disrupted phagosome-lysosome fusion.

In keeping with this, we also observe V8 cleavage of pyruvate kinase PKM2, a glycolytic intermediate critical for ROS production (41). ROS are potent effectors of antimicrobial activity within the neutrophil phagolysosome, with the ability to elicit oxidative stress and DNA damage of the engulfed microbe (42). Demonstrating this, an *in vitro* study investigating the effects of PKM2 inhibition or deletion reported an increase in survival of *S. aureus* stemming from suppressed ROS production (41). Notably, PKM2 was also identified as a V8 substrate in our human neutrophil study (23), bolstering the implication of metabolic disruption and ROS ablation as a mechanism of V8-mediated immune modulation. Across both studies, V8 processing of PKM2 occurred in the A domain that controls dimer formation (43). Moreover, the binding pocket of PKM2 is located at the interface of domains A and B, hence cleavage here may serve to inhibit substrate binding for PKM2. As with our human neutrophil study (23), we also observed continued targeting of ROS production by V8 in our lung data set. Herein, we noted cleavage of the delta type (δ) of protein kinase C (PKC), while we previously reported proteolysis of the alpha type of this protein in the neutrophil setting (23). PKCδ can regulate the NADPH oxidase complex, the central enzyme for ROS production, whereby patients with a deficiency in this protein present with reduced levels of oxyradicals and suffer from CGD-like symptoms (44–46). Beyond PKCδ and PKM2, TAGS-CR also captured a variety of other ROS modulators cleaved by V8, including Ras homolog family member C, Prenylcysteine oxidase 1, RAP1B, and Centaurin-delta-2 (Tables S1 to S3).

While the defense mechanisms described above are fundamental to pathogen clearance, neutrophils must first reach the site of infection to execute their antimicrobial capabilities. In a process known as leukocyte extravasation, prompted by cytokines during microbial infection, neutrophils first become arrested to the cell surface, followed by adhesion, activation, and finally, diapedesis through the endothelium (47). IPA identified 15 V8 substrates belonging to this signaling pathway, and, while these included several actin molecules (48), as well as GPCR interactors such as guanine nucleotide-binding protein G(i) subunit alpha-2 (49), perhaps the most relevant to this biological process were the integrin and integrin-interacting molecules (Table S3) (50). For example, V8 cleaves intercellular adhesion molecule 1 (ICAM1), which is an important receptor molecule that binds to the lymphocyte function antigen-1 (LFA-1), an integrin dimer that promotes immune cell arrest (50). Of notable interest, TAGS-CR previously captured the integrin alpha-L subunit of LFA-1 as a constituent of the neutrophil V8 pathodegradome. Collectively, our combined data suggest that V8 may interfere with both sides of this interaction, dismantling this key neutrophil-recruiting complex. This notion is further bolstered by our finding that V8 mutants of *S. aureus* have impaired survival during engagement with human neutrophils (23).

Relevant to the LFA-1/ICAM-1 complex, our N-terminomic data also identified integrin molecules responsible for neutrophil activation as being modulated by V8. This included kindlin-3, responsible for “inside out signaling” that activates LFA-1 prior to ICAM-1 binding (51). Patients with mutations in the kindlin-3 encoding *FERMT3* gene present with ablated neutrophil migration, which is known as leukocyte adhesion deficiency-III (LAD-III) (51). In human lung tissue, we observed V8 cleavage of this molecule at position 500 corresponding to this protein’s key binding site, the FERM domain, that facilitates attachment to its interacting partner, the LFA-1 beta subunit (51, 52). Yet again, we also identified kindlin-3 as a substrate in our V8-neutrophil study, capturing the same cleavage site of position 500 in the FERM domain (23). Additionally, this cleavage site is close to the position of the mutated amino acid (552) that results in LAD-III, highlighting a possible role for V8 in mimicking this effect. Importantly, we were able to validate V8 cleavage of this molecule via western blot analysis (Fig. 3B). Here, we captured full-length protein at ~75 kDa in untreated control lanes alongside likely modified forms of the protein, given how heavily it is phosphorylated (Fig. 3B) (53). Furthermore, this protein has also been observed to undergo endogenous processing by calpain, with cleavage observed at position 373 (54) (Fig. 3B). Given that the calpain small subunit 1 also appeared as a V8 substrate within our data set, we can deduce that this protein is present and active within the host. Together with calpain cleavage, V8 proteolysis of kindlin-3 at aa 500 would result in a fragment of approximately ~14 kDa, directly in line with the fragments observed in V8-treated lanes (Fig. 3B).

**Fig 4:**
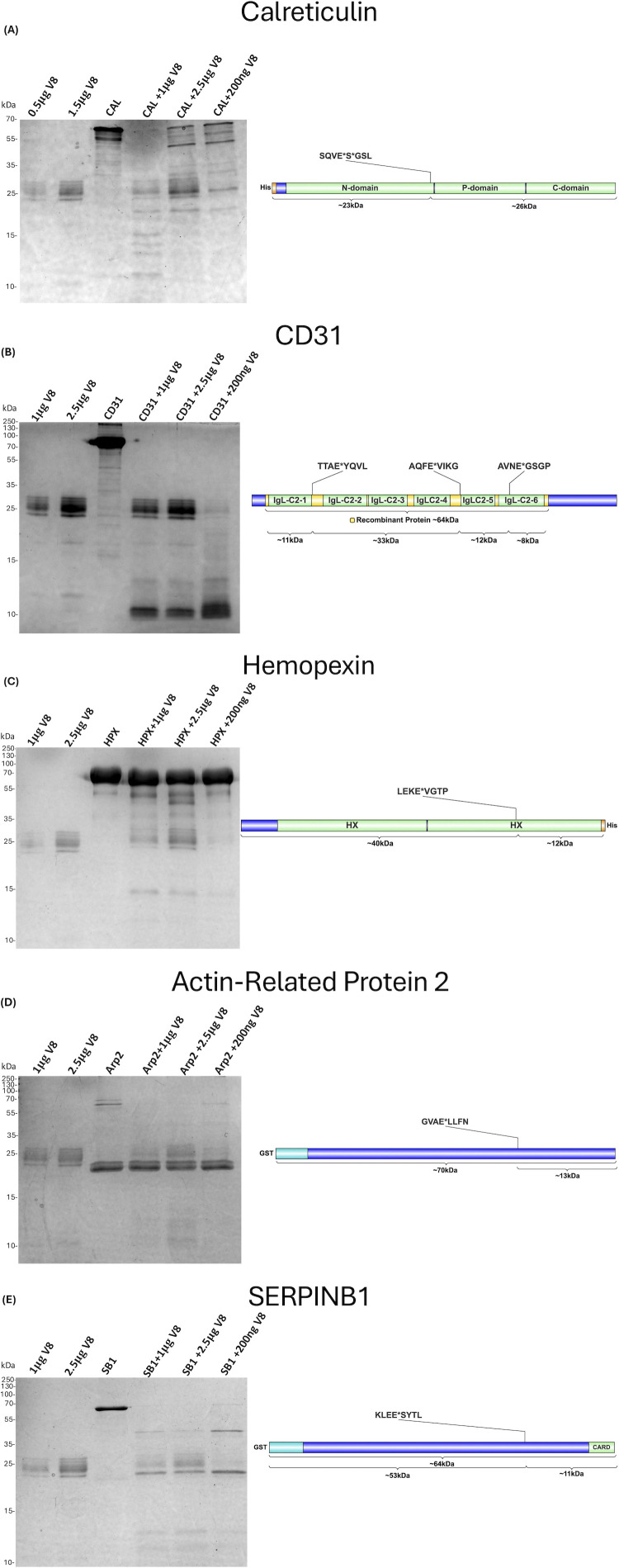
Validation of human lung targets for the V8 protease using purified proteins. Recombinant proteins of (**A**) Calreticulin, (**B**) CD31, (**C**) Hemopexin, (**D**) Actin-Related Protein-2, and (**E**) SERPINB1 all underwent treatment with V8 protease. Purified V8, alongside the untreated purified protein, was included as a control. In the event a truncated protein was purchased, this fragment is highlighted in yellow in the relevant panel. CAL = Calreticulin. IgL-C2 = Immunoglobulin-like C2 domain. HPX = Hemopexin. HX = Hemopexin-like domain. Arp2 = Actin-related protein 2. SB1 = SerpinB1. CARD = Card Domain.

Continued overlap with our V8-neutrophil data set (23) is seen herein with cleavage of calreticulin. This calcium-binding chaperone serves as a multifunctional protein found in the endoplasmic reticulum as well as on the neutrophil cell surface (55, 56). Previous studies have highlighted the antimicrobial role of this protein in promoting *S. aureus* phagocytosis via its pathogen-binding abilities (56). Additionally, calreticulin can also contribute to ROS production either through stabilization of production intermediates or in response to certain synthetic antimicrobial peptides (55, 57). Herein, a neo-N-termini for calreticulin at position 193 was captured via TAGS-CR (Table S2). This cleavage site is found in the P-domain, representing the main site of chaperone activity (58, 59). We validated cleavage of this protein via Coomassie staining of purified protein digested with V8. Here, we reveal degradation products slightly below 25 kDa that are directly in line with the predicted product from our N-terminomic data (Fig. 4A). As a means of secondary validation, we performed in-gel digestion of the excised bands at this molecular weight, followed by mass spectrometry. In so doing, we positively identified the same start position belonging to a V8 semi-tryptic peptide captured following V8 treatment (Table S4). Finally, we were able to validate V8 cleavage of platelet endothelial cell adhesion molecule 1 or CD31, a critical membrane protein also found to be necessary for neutrophil migration. This V8 substrate localizes at the uropod of migrating neutrophils and activates integrin signaling (60). Following SDS-PAGE and in-gel digestion of the purified protein (Fig. 4B), MS/MS analysis was performed, and the corresponding TAGS-CR cleavage sites were detected at positions 512 and 421 (Table S5).

### V8 likely exerts influence over host-pathogen dynamics through a role in iron acquisition

Iron’s key importance in many cellular processes underlies the constant competition between microbes and their host for this essential nutrient (61, 62). Importantly, the delicate interplay between host and pathogen iron acquisition has been well documented to influence infection progression and outcome (61, 62). While humans sequester iron for their own benefit, to prevent intracellular toxicity and mitigate bacterial acquisition, a phenomenon termed nutritional immunity, pathogenic bacteria like *S. aureus* have evolved various mechanisms geared toward amassing vertebrate iron (61, 62). In iron-deficient environments, *S. aureus* scavenges iron from iron-complexed molecules such as transferrin and lactoferrin using small-molecule siderophores such as staphyloferrin A and staphyloferrin B (61, 62). Furthermore, this bacterium also possesses the iron-regulated surface determinant (Isd) system that is highly specialized for heme uptake and utilization from hemoglobin or haptoglobin–hemoglobin complexes (61, 62). Herein, we observe a possible alternative pathway by which *S. aureus* may liberate free iron for its utilization via the proteolytic activity of the V8 protease. In our data set, we captured neo-N-termini of two types of iron-binding transferrins, lactotransferrin, and serotransferrin (Tables S1 and S2). In this regard, it is possible that staphyloferrin A and B may not work in isolation to obtain iron from complexed transferrin but are aided by the V8 protease. Transferrin molecules consist of two lobes, the N-lobe and C-lobe, that each fold to form an iron-binding cleft (63). We captured cleavage of serotransferrin at position 523 and cleavage of lactotransferrin at position 218, corresponding to their C-lobe and N-lobe, respectively. Proteolysis at these positions may mimic protein unfolding and/or the conformational change that occurs when these molecules release iron in low pH conditions (63). In keeping with V8 targeting of host iron-complexed proteins, our data also highlighted V8 cleavage of three of the four hemoglobin subunits, specifically alpha, beta, and delta. In the context of *S. aureus* infections, cleavage of hemoglobin by V8 may serve to accelerate the uptake of free heme and enhance bacterial survival and proliferation.

In addition, our data also showed cleavage of another iron-binding protein, hemopexin, known to form a complex with free heme to prevent microbial utilization. Interestingly, it has been demonstrated *in vitro* that *S. aureus* cannot exploit this host molecule for its own iron gain, as hemopexin is seemingly unrecognizable by the Isd system (64, 65). In this regard, cleavage of hemopexin by V8 may represent an alternative strategy for *S. aureus* to access this previously untapped source of iron. V8-mediated proteolysis of this molecule occurs at position 349 found in the C-terminal hemopexin-like domain. This host iron source contains two hemopexin-like domains, the other located at the N-terminus and is homologous in structure. These fold together to form a heme binding pocket, similar to that of transferrin molecules (66). Thus, V8 cleavage within any domain, such as the C-terminus, may result in exposure of bound heme for *S. aureus* utilization. We were able to validate V8 cleavage of hemopexin via Coomassie staining of V8-digested purified recombinant protein (Fig. 4C). Following in-gel digestion and MS/MS analysis, we captured a V8 semi-tryptic peptide with start position 349 that is identical to our N-terminomic data (Table S6).

Beyond iron-complexed proteins, IPA analysis also highlighted various V8 targets involved in iron homeostasis (Table S3). These include cytoplasmic aconitate hydratase or iron regulatory protein 1 that possess RNA binding capabilities necessary for coordinating iron transport, storage, and utilization (67). TAGS-CR captured cleavage of this protein at aa 520 located in domain 3 that together with domains 1 through 4 forms the binding pocket necessary for contact with iron regulatory elements (68, 69). Truncated forms of this protein without all of the necessary domains have been predicted to lose RNA-binding capacity (68). Other V8 substrates found to be involved in regulatory control of iron levels include ferroxidase (CP), heme oxygenase 1 (HMOX1), and Cullin-associated NEDD8-dissociated protein (CAND1) (Tables S1 to S3).

### TAGS-CR uncovers a potential role for V8 in epithelial barrier disruption

The protective interface of human epithelial barriers, such as that of the lungs, shields the internal environment of the body from harmful external agents, including allergens, pollutants, toxins, and importantly, invading pathogens like *S. aureus* (70, 71). Disruption of barrier integrity has been highlighted as a critical mechanism of action in staphylococcal infections, with several virulence factors disrupting barrier function, including alpha toxin, enterotoxin-like superantigen SE/Q, exfoliative toxins, phenol-soluble modulins, leukocidins, and indeed the extracellular proteases (17, 72–75). Junctional complexes, including tight and adherens junctions, are notably vulnerable to pathogen manipulation, which compromises barrier integrity and increases host susceptibility to invasion and infection. (76, 77). In a previous study, V8 has been shown to specifically induce airway epithelial barrier destruction via alterations in tight junction protein levels, such as that of zona occludens-1 or ZO-1 (16). Indeed, fundamental to tight junctions are transmembrane proteins such as ZO-1, ZO-2, or ZO-3 that form cell-to-cell complexes, and actin proteins that help anchor these structures (16). Herein, we present supporting evidence that V8 not only cleaves the transmembrane proteins of zona occludens, but furthermore the supporting actin proteins (Tables S1 and S2).

TAGS-CR captured V8 cleavage of a tight-junction protein 2 (ZO-2) isoform at three positions: 299, 306, and 310. These cleavage sites all correspond to the PDZ-3 domain, one of three PDZ domains shown to be important for protein-protein interactions mediating the assembly of tight junction complexes (78, 79). While each PDZ domain has been shown to have different binding partners, PDZ-3 can bind to the junction adhesion molecule or JAM, an interaction that facilitates proper barrier function (78, 80). Interestingly, our data also showed that PDZ-associated proteins are targeted by V8, representing a multifaceted point of proteolytic interference where V8 can act on both sides of this junctional complex. To this effect, we recorded cleavage of alpha-actinin-4, shown to interact with the PDZ-1 domain of ZO-1 (81). V8 cleavage was captured at positions 480 and 483, located within the second spectrin repeat, a three-helix structure that, in combination with the other remaining spectrin repeats, forms a rod domain that is fundamental to maintaining stable interactions (82, 83). Alongside alpha actinin-4, alpha actinin-1 was also cleaved by V8 at positions 461 and 464, similarly found in a spectrin 2 repeat, thus highlighting a conserved structural vulnerability to bacterial proteases among alpha actinin isoforms.

Beyond this, we recorded cleavage of numerous supporting proteins that maintain both tight and adherens junctional integrity. These included Plectin, a vital cytoskeletal linker whose deficiency has been shown to distort both junction types (84). We additionally found multiple constituents of the hexameric non-muscle myosin-II molecule, a motor protein responsible for generating tension between actin filaments and maintaining cell-to-cell contact, to be cleaved by V8 (Tables S1 and S2). Every heavy chain isoform of this molecule was identified as a V8 target, including myosin-9, myosin-10, and myosin-14, which encode the protein products NMHC IIA, NMHC IIB, and NMHC IIC, respectively (Tables S1 and S2). Previous studies have highlighted the importance of myosin-9 and myosin-10, where knockout and knockdown mice, respectively, both demonstrated defects in junction formation (85–87). In keeping with the cleavage of tight junction supporting molecules by V8, TAGS-CR also identified Filamin-A as an additional protease substrate (Tables S1 and S2). We captured 9 cleavage events across this 2,647 amino acid molecule (Table S2), although even one site of proteolysis on this protein is likely to inhibit actin filament cross-linking. As previously mentioned, V8 can promote the release of bradykinin, and interestingly, this molecule works primarily to disrupt the epithelial barrier by promoting the phosphorylation of Filamin-A, resulting in a reduction of actin cross-linking and consequently, barrier damage (88). Thus, in combined efforts with bradykinin, our data show an alternative route of how V8 could dismantle the actin cytoskeleton via Filamin-A cleavage, causing loss of junctional integrity.

Finally, actin-related protein-2, a critical component of the Arp2/3 complex, which similarly promotes actin nucleation and junction assembly (89), was cleaved at position 281 by V8. Given that V8 cleavage occurs in subdomain 3 of Arp2, which together with subdomain 4, forms the binding cleft for nucleotide interactions (90), cleavage here may cause structural defects and prevent actin polymerization. We validated this cleavage event first via Coomassie staining, where V8-treated purified Arp2 protein showed the expected 13 kDa fragment predicted by our N-terminomic data (Fig. 4E). We then performed an in-gel digest of the excised bands at this molecular weight and subjected them to MS/MS analysis. In so doing, we captured a V8 semi-tryptic peptide with the TAGS-CR mapped start position of 281, thus validating our findings (Table S7).

### Cleavage by the V8 protease likely modulates host inflammatory pathways

Inflammation is a central hallmark of *S. aureus* pulmonary infections, and, indeed, several *S. aureus* virulence factors have recognized roles in how they orchestrate this hyperinflammatory response (19, 91–96). Herein, our data suggest that V8 cleavage of key anti-inflammatory proteins and other immune regulators allows it to function in concert with these other virulence factors to exacerbate inflammation by *S. aureus* within the human lung.

Among these are members of the Annexin A family. These are diverse multifunctional proteins, with representatives such as Annexin A1 and A2 that have predominant roles in the resolution of inflammation, cleaved by V8 in our studies (97, 98). Targeted degradation of Annexin A2 at multiple positions by V8, including amino acids 54, 150, and 157 (Table S2), may lead to functional inactivation and decreased molecular abundance. Previously, downregulation of Annexin A2 has been shown to result in NLRP inflammasome activation and subsequent increase in pro-inflammatory cytokine production (99). In a similar vein, V8 cleavage of Annexin A1 may also lead to detrimental consequences for the host by exacerbating the inflammatory response. The anti-inflammatory nature of this molecule stems from its regulation of neutrophil migration and cytokine release as well as modulation of other immune cell types (97, 100–102). Annexin A1 functions not only to reduce immune cell migration and promote apoptosis by functioning as an “eat me” signal, but furthermore, it can also regulate cytokines such as TNF-α and IL-6, as well as modulate macrophage function, leading to immunosuppression (97, 100, 101). TAGS-CR captured V8 cleavage of this protein at position 63, found within the first C-terminal calcium binding repeat motif (102), potentially liberating a ~32 kDa fragment. Interestingly, a previous study investigating calpain cleavage of this protein found that the resulting 33 kDa C-terminal fragment from that cleavage event has a proinflammatory effect via activation of endothelial ERK1/2 signaling, which is essential for neutrophil migration (103). Thus, V8 cleavage may mimic this effect by generating a fragment of Annexin A1 of similar size to strategically enhance neutrophil infiltration and tissue damage.

Beyond the annexin family of proteins, other biomarkers of inflammation vulnerable to V8 proteolytic activity include gelsolin, found to be cleaved at position 469 (Tables S1 and S2). The immunosuppressive effect of this molecule stems from its ability to function as a sink for pro-inflammatory molecules (104–106). During cellular injury, F-actin is released from damaged cells and functions as a “damage-associated molecular pattern” or a DAMP, known to initiate an immune response (107). As an actin scavenger, gelsolin can suppress the effects of F-actin, a known culprit for potentiating inflammation in adult respiratory distress syndrome (ARDS) (104). Given that *S. aureus* is a frequent cause of bacterial ARDS, where hallmarks include endothelial barrier damage and permeability, as well as acute inflammation (108), cleavage here implicates V8 in exacerbating pulmonary immune dysregulation. Another dimension of gelsolin’s function as a pro-inflammatory sink is its ability to bind to *S. aureus* lipoteichoic acids (LTAs) (104). The effects of gelsolin binding to LTA include inhibition of NF-κB translocation and dampened neutrophil activation and cytokine release (104). Thus, V8 seemingly undermines the multifaceted ability of gelsolin to strategically control the immune response, promoting a pro-inflammatory environment that can lead to poor infection outcomes.

Alongside the annexin molecules and gelsolin, TAGS-CR captured V8 cleavage of other critical immune regulators such as several immunoglobulin chains, macrophage-capping protein (CAPG), and proteins involved in MHC class I and II antigen presentation, including proteasome subunit beta type-9 and HLA class II histocompatibility antigen (HLA-DRB4) (Tables S1 and S2). In particular, the proteasome subunit beta type-9, also known as LMP2 or PSMB9, part of the ubiquitin-proteasome system, fundamental for protein processing and homeostasis, has a pivotal role in the control of inflammation (109, 110). While the biological role of this protein is complex, previous research has documented an amino acid substitution in PSMB-9 as resulting in proteasome dysfunction, consequently giving rise to “proteasome-associated autoinflammatory syndrome (PRAAS)” (109). Moreover, proteasome defects can induce cellular distress and trigger the unfolded protein response, leading to activation of inflammatory signaling cascades (109). Thus, V8 may mimic this effect and compromise PSMB-9 function, inducing cellular stress and aberrant inflammatory signaling, presenting a novel mechanism for immune manipulation.

Meanwhile, V8 cleavage of immune targets like the major vault protein (MVP), otherwise known as lung resistance-related protein, could have specific implications for lung tissue defense (Table S1). This protein can be recruited to lipid rafts after bacterial infection, with the overall effect of mediating pathogen entry (111, 112). While this molecule has been investigated in the context of *Pseudomonas aeruginosa* infections and has even been observed for viral infections with Influenza A, our discovery of this protein as a V8 target could indicate a similar role for this protein during *S. aureus* infections. While cleavage of MVP may prevent bacterial uptake, other implications of MVP cleavage could include dysregulation of immune signaling (112). In support of this, the MVP has also been found to represent a biomarker for inflammation, and it is hypothesized that increased MVP levels may function to reduce the inflammatory response—hence V8 may trigger the opposite effect by potentially degrading this protein (113).

Finally, we noted the cleavage of a known regulator of inflammation, SERPINB1, that functions as both a checkpoint for the production of pro-inflammatory caspases during microbial infections and as a host serine protease inhibitor (114, 115). In regard to the latter, neutrophil viability has been shown to suffer in the event these immune cells are no longer protected from host proteases as a result of SERPINB1 deficiency (114). In fact, gene knockouts of SERPINB1 have been investigated in the context of *P. aeruginosa* lung infection, whereby deficiency in mice results in heightened protease activity, degradation of surfactant protein-D, consistent cytokine production, and increased mortality (115). This previous study also highlighted how exogenous administration of SERPINB1 to these mutant mice restored normal levels of bacterial clearance (115). TAGS-CR captured V8 cleavage of this molecule at position 281, upstream of the reactive center loop (RCL), where protease binding occurs (113). Notably, it is the conformational change of the SERPINB1-protease complex after binding that neutralizes the activity of the bound enzyme (116). This process involves the RCL’s N-terminal segment becoming embedded into the beta sheet A of SERPINB1, where, importantly, amino acid 281 is located (116–118). Given that this sequence of events stabilizes the SERPINB1 molecule, V8 cleavage here may function as a regulatory mechanism that modulates its inhibitory capacity during infection, with the overall effect of perpetuating inflammation. MS/MS analysis following in-gel V8 digestion of purified SERPINB1 (Fig. 4D) detected a V8 semi-tryptic peptide with the TAGS-CR identified start position of amino acid 281 (Table S8).

### Concluding remarks

Pathodegradome mapping is a highly powerful approach that provides direct insight into proteolytic-driven modulation of cellular networks within living systems. For harmful human pathogens like *S. aureus* that possess an effective repertoire of secreted proteases, substrate mapping has become foundational to understanding how they elicit infection. Given the sheer breadth and diversity of *S. aureus* diseases, how they manifest can vary in the human body. Furthermore, each host niche presents unique and differing immune challenges that this bacterium must overcome. Herein, we present substrate mapping for V8 that presents insight into mechanisms of immune response and pathogen clearance, with direct relevance to the human lung environment. For example, we demonstrate cleavage of Complement C3 and the MVP—both of which are suggested from previous studies (34, 111, 112) to provide specific innate immune protection in the lungs. We also identify several V8 host substrates important for the proper formation of tight and adherens junctional structures, providing an explanation for the previously described role for V8 as a disruptor of epithelial barrier integrity (16). Specifically, TAGS-CR captured V8 cleavage of zona occluden ZO-2 and alpha actinin molecules, which are foundational to these epithelial barrier complexes. Moreover, we also detected cleavage of supporting actin molecules such as Plectin, constituents of the Hexameric non-muscle myosin-II molecule, Filamin-A, as well as the actin-related protein-2.

Our lung degradome also contains numerous areas of support and confirmation for recent work by our group studying how V8 manipulates neutrophil function (23). Herein, we identified several targets for V8 that overlap in human lungs and neutrophils, including pyruvate kinase PKM2, kindlin-3, and calreticulin. Moreover, we highlight dual-sided interactivity of V8 with certain neutrophil associations, such as ICAM-1-LFA-1, with the former identified herein, while the latter was identified in our work with human neutrophils. Coupled with this, we observed targeting of the same neutrophil complex in both pathodegradomes—the NADPH molecule—however, V8 cleaved different constituents in these separate conditions.

We also reveal new dimensions for V8’s pathogenic ability by uncovering previously undefined substrates. These included iron-binding and iron-complexed host proteins, suggesting an unrecognized role of the extracellular proteases in disrupting nutritional immunity and iron homeostasis. In addition, we present a role for V8 in potentiating pro-inflammatory signaling, with the likely overall effect of eliciting tissue damage and immune dysregulation. Specifically, we highlighted cleavage of several molecules functioning as pro-inflammatory sinks together with other important regulators of immune function.

While our *in vitro* data strongly suggest that SspA activity could influence the course of respiratory infection, currently, no *in vivo* model has been performed to test this. However, previous studies have demonstrated attenuation of the V8 mutant in other relevant animal models of infection, such as an abscess model, an intravenous infection model, a burn wound model, and a murine model of itch analysis (3, 18, 119). Collectively, these data support the hypothesis that V8 may be equally relevant in the context of *S. aureus* lung infection, pointing to a logical and impactful next step for this line of research. Moreover, an *in vivo* pneumonia mouse model could further be supported by *ex vivo* human studies investigating the activity of V8 in AECs.

Collectively, the application of TAGS-CR has enabled us unprecedented access to V8’s substrate repertoire, revealing novel molecular targets and mechanistic insights that were previously not feasible with dated conventional approaches. The critical nature of this study is that it expands our knowledge of how this protease subverts the host immune system, thereby revealing vulnerabilities in protease–host interactions that could be exploited for therapeutic intervention. Beyond the lung environment, deployment of TAGS-CR to other host niches promises to make significant strides in mapping protease-substrate networks, thereby advancing our broader understanding of host-pathogen dynamics.

## MATERIALS AND METHODS

### Human lung proteome treatment with the V8 protease

Human lung tissues (0.5 g) were purchased from BioIVT. Tissue was divided into six equal portions, equating to approximately 0.083 g, and added to tissue lysis tubes (Navy Lysis Kit, Next Advance) with 1 mL of the Tissue Protein Extraction Reagent T-Per (ThermoFisher Scientific). Homogenization was performed using a bench-top tissue homogenizer, with lysis proceeding for a total of 4 min. Homogenates were pooled and quantified using the Pierce 660 nm Protein Assay (ThermoFisher Scientific). Human lung proteomes were standardized to 1 mg/mL and incubated ±200 ng of V8 protease (MilliporeSigma), at 37°C for 16 h. Following protease treatment and overnight incubation, dry guanidinium hydrochloride was added to a final concentration of 6M to neutralize protease activity.

### N-terminomic processing: TAGS-CR and mass spectrometry

N-terminomic experimentation and mass spectrometry were performed as previously described in Mustor et al. (23), following protease treatment.

### Data analysis

Data analysis was performed as outlined in Mustor et al. (23) with the following modification: Each experiment was injected into the mass spectrometer once to give *n*=3 for both the control group and the V8 protease treatment condition.

### SDS-PAGE, immunoblotting, and Coomassie staining

SDS-PAGE, immunoblotting, and Coomassie staining were all performed as described in Mustor et al. (23), with the only variation being the identity of the primary antibodies and recombinant proteins utilized. In this study, primary antibodies used were Complement C3 (abcam, ab200999) and Kindlin-3 (ThermoFisher Scientific, #PA5-30847). Recombinant proteins were Calreticulin (abcam, ab91577), CD31 (ThermoFisher Scientific, #150-06-100UG), Hemopexin (ThermoFisher Scientific, #50-162-1668), SERPINB1 (Abnova, #H00001992-P01), and actin-related protein-2 (abcam, ab217837).

### In**-**gel digestion

In-gel digestion was performed as described in Mustor et al. (23).

## Data Availability

Two submissions of raw mass spectrometry data were deposited to the ProteomeXchange consortium via the PRIDE repository. Human lung N-terminomic data can be found under the DatasetData set ID PXD070241, while in-gel digestion results for Ccalreticulin, CD31, Hemopexin, SERPINB1, and Aactin-Rrelated Pprotein-2 can be found under the ID PXD07024197.

## SUPPLEMENTAL MATERIAL

Fig. S1 iai.00725-25-s0001.tiff ICELOGO plots comparing V8 activity across various infection relevant sources.

Supplemental tables iai.00725-25-s0002.xlsx Tables S1 to S8.

ASM does not own the copyrights to Supplemental Material that may be linked to, or accessed through, an article. The authors have granted ASM a non-exclusive, world-wide license to publish the Supplemental Material files. Please contact the corresponding author directly for reuse.
