# Supplementary figures and images for: Defining the human lung pathodegradome of the V8 protease from Staphylococcus aureus
Source: Infect Immun. 2026 Mar 5;94(4):e00725-25. doi: 10.1128/iai.00725-25 (PMC13081733; doi:10.1128/iai.00725-25)

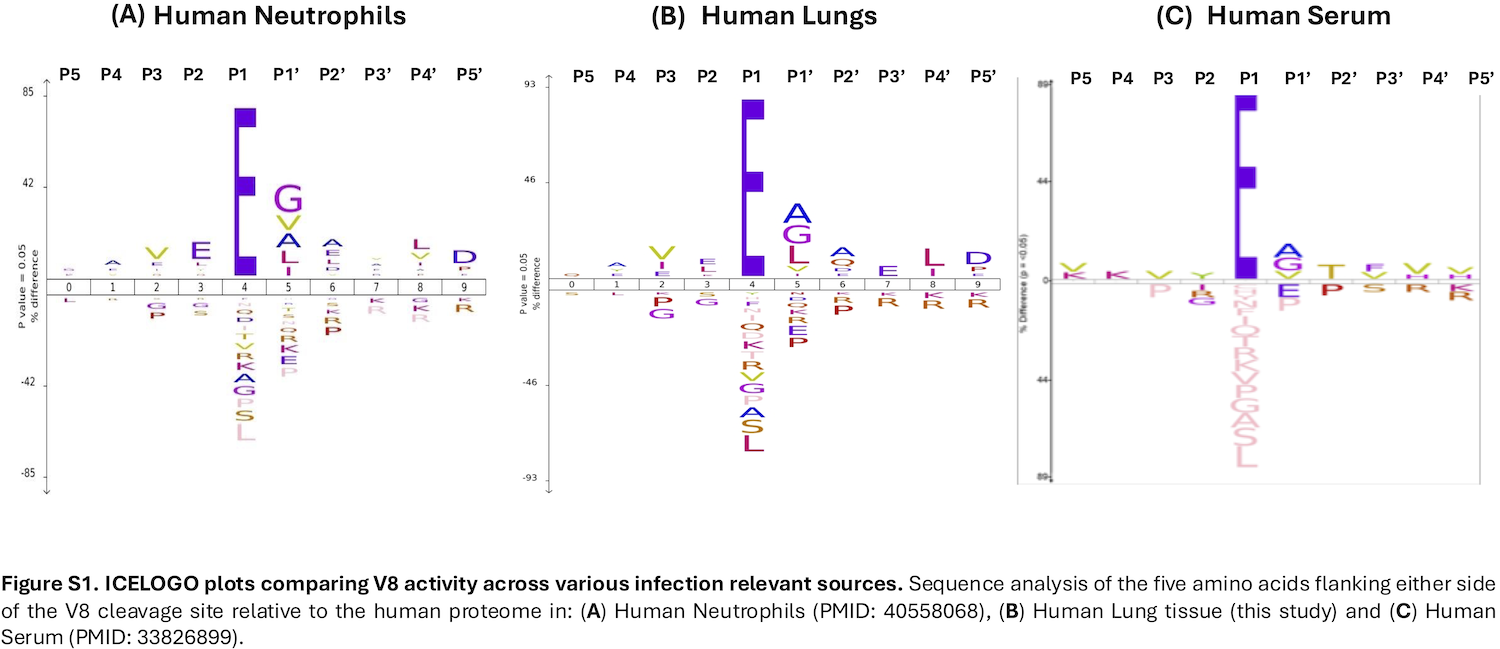

Supplement: Fig. S1 — ICELOGO plots comparing V8 activity across various infection relevant sources. [file iai.00725-25-s0001.tiff]
